# A Cuproptosis Nanoparticle Reverses Lung Adenocarcinoma Drug Resistance

**DOI:** 10.1002/exp2.70250

**Published:** 2026-11-10

**Authors:** Leilei Wu, Kun Li, Han Wang, Shangshang Ma, Rangrang Wang, Yongfei Fan, Xichun Qin, Mingjun Li, Diego Gonzalez‐Rivas, Xiuru Ji, Dalong Ni, Dong Xie

**Affiliations:** ^1^ Department of Thoracic Surgery Shanghai Pulmonary Hospital School of Medicine Tongji University Shanghai P. R. China; ^2^ Department of Thoracic Surgery Zhejiang Cancer Hospital Hangzhou Institute of Medicine (HIM) Chinese Academy of Sciences Hangzhou P. R. China; ^3^ Department of Orthopaedics Shanghai Key Laboratory for Prevention and Treatment of Bone and Joint Diseases Shanghai Institute of Traumatology and Orthopaedics Ruijin Hospital Shanghai Jiao Tong University School of Medicine Shanghai P. R. China; ^4^ Department of Thoracic Surgery and Minimally Invasive Thoracic Surgery Unit Coruña University Hospital Coruña Spain; ^5^ Suzhou Institute of Biomedical Engineering and Technology Chinese Academy of Sciences Suzhou P. R. China

**Keywords:** cuproptosis, *KRAS* mutation, lung adenocarcinoma, nanomedicine, targeted therapy

## Abstract

Residual tumors that received targeted therapy often evolve into drug resistance, with an increasing risk of recurrence and metastasis. By analyzing patient data, it is found that *KRAS*‐mutated lung adenocarcinoma (LUAD) exhibits a worse survival rate than wild‐type LUAD. Although the clinical drug AMG‐510 has been approved to treat *KRAS(G12C)*‐mutated LUAD, its effectiveness is still limited by drug resistance. Cuproptosis, a newly identified mode of cellular death, shows promise in cancer treatment and in reversing drug resistance. By comparing cell lines with different sensitivities to AMG‐510, we discovered that *ATP7B*, a key regulator of cuproptosis, was up‐regulated in AMG‐510‐resistant cell lines to decrease their sensitivity to cuproptosis. Therefore, a targeted sensitization strategy focused on cuproptosis was proposed using a single nanoparticle based on bovine serum albumin encapsulating copper ions (termed CuNPs). Both in vitro and in vivo results demonstrated that these nanoparticles released copper ions in an acidic environment, and the following CuNP‐triggered cuproptosis could effectively down‐regulated *ATP7B*, which successfully overcame AMG‐510 resistance with a synergistic antitumor effect. Collectively, targeting cuproptosis in KRAS‐mutated lung adenocarcinoma emerges as a compelling strategy to address drug resistance.

## Graphical abstract

Copper‐based nanoparticles offer a promising strategy to improve treatment for *KRAS*‐mutated lung adenocarcinoma. These nanoparticles release copper ions in acidic environments, triggering copper‐dependent cell death and reducing *ATP7B* expression. When combined with the KRAS inhibitor sotorasib (AMG‐510), they enhance anticancer activity in drug‐insensitive lung cancer cells and suppress tumor growth in mice.

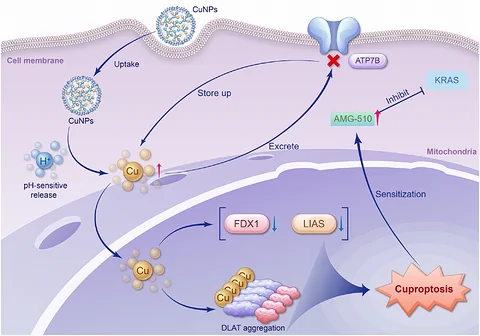

## 1 Introduction

Lung cancer remains the leading cause of malignancy‐related deaths worldwide. Non‐small cell lung cancer (NSCLC) constitutes approximately 80%–85% of all lung cancer cases, with lung adenocarcinoma (LUAD) being the predominant histological type [1, 2, 3]. Unfortunately, the prognosis of LUAD is not satisfactory in clinical practice [4]. Multiple factors influence the survival outcome of LUAD patients, including disease stage, driver gene mutations‐such as epidermal growth factor receptor (*EGFR*) and Kirsten rat sarcoma viral oncogene (*KRAS*)‐and treatment strategies [5, 6, 7, 8]. In the Chinese NSCLC population, the *KRAS* mutation rate is approximately 11.7%, although data from the Catalogue of Somatic Mutations in Cancer (COSMIC) database indicate that this rate may be as high as 17.3% [9, 10]. Historically, chemotherapy has been the mainstay of systemic treatment for patients harboring *KRAS* mutations, yet the survival benefits have been limited [7]. The limited efficacy of chemotherapy is partly attributable to the molecular characteristics of KRAS. KRAS protein was long considered “undruggable” due to the absence of a suitable binding for small‐molecules inhibitors [11, 12]. However, in 2013, *Ostrem* et al. identified a hydrophobic pocket in the KRAS(G12C) mutant protein that allows binding small molecules in an inactive state [13, 14]. This discovery paved the way for the development of AMG‐510, the first KRAS(G12C) inhibitor [15]. Clinical trials of AMG‐510 in NSCLC patients demonstrated an objective response rate of only 32.2% [16]. Despite this modest efficacy, the U.S. Food and Drug Administration (FDA) approved AMG‐510 in 2021 for the subsequent treatment of NSCLC patients with *KRAS(G12C)* mutation [17]. However, a phase III randomized controlled clinical trial (NCT04303780) conducted in 2022 comparing AMG‐510 with docetaxel in *KRAS(G12C)*‐mutated NSCLC failed to show an overall survival (OS) advantage, although progression‐free survival (PFS) was prolonged [18, 19]. These findings suggest that AMG‐510 monotherapy offers limited clinical benefit. Therefore, there is an urgent need to explore AMG‐510‐based combination therapies or alternative treatment strategies to improve survival outcomes in LUAD patients harboring *KRAS(G12C)* mutations.

Recently, cuproptosis has been identified as a novel form of regulated cell death, distinct from other known pathways such as ferroptosis and apoptosis [20, 21, 22]. This mechanism involves the direct binding of copper to fatty acylated components within the tricarboxylic acid cycle, triggering proteotoxic stress and ultimately cell death. The aberrant copper interaction leads to the aggregation of fatty acylated proteins and the depletion of iron–sulfur cluster proteins. Inhibiting mitochondrial respiration through pharmacological intervention has emerged as a potential strategy for treating diseases associated with cuproptosis. Notably, LUAD cells express a high abundance of lipoylated mitochondrial proteins and depend heavily on copper‐mediated aerobic respiration [20, 21]. This metabolic reliance suggests that targeting copper homeostasis could offer a novel therapeutic avenue for LUAD. In line with this metabolic reliance, previous studies over the past decades have also highlighted the anticancer potential of copper complexes [23, 24]. Copper has been shown to promote cancer cell death via apoptosis and/or the accumulation of free radicals [25]. These insights into cuproptosis opened new possibilities for LUAD treatment. One study evaluated cuproptosis‐related genes in LUAD, revealing their prognostic significance and association with drug sensitivity, thereby underscoring the intrinsic link between cuproptosis and LUAD pathogenesis [26]. Furthermore, another investigation demonstrated that copper‐containing biomaterials capable of inducing cuproptosis effectively suppressed tumor growth in bladder cancer models [27]. Taken together, cuproptosis represents a promising and innovative therapeutic target for LUAD, warranting further exploration in both preclinical and clinical settings.

A wide range of organic, inorganic, and hybrid nanomaterials have been applied in cancer therapy owing to their favorable biosafety and significant therapeutic potential [28, 29, 30, 31, 32, 33]. Recent findings have also revealed that cuproptosis can enhance anti‐tumor immunity to sensitize therapeutic effect of radiotherapy [34, 35, 36]. Therefore, developing a copper‐based nanomaterial capable of inducing cuproptosis in *KRAS(G12C)‐*mutated LUAD or enhancing the sensitivity of *KRAS(G12C)*‐mutated LUAD to AMG‐510 is a worthwhile endeavor. Given this hypothesis, we first examined clinical data from 3251 cases at Shanghai Pulmonary Hospital, which revealed that patients harboring *KRAS* mutations exhibited significantly poorer prognosis than those with wild‐type *KRAS*. Besides, Cu‐based nanoparticle composed of bovine serum albumin (BSA) encapsulating copper ions (termed CuNPs) was developed for the treatment of *KRAS*‐mutated LUAD. Initial demonstrations showed that CuNPs released copper ions in an acidic environment, suggesting targeted enrichment in tumor regions and induction of cuproptosis in tumor cells. Our study demonstrated that CuNPs‐triggered cuproptosis effectively inhibits LUAD proliferation and metastasis both in vivo and in vitro. Notably, transcriptomic analysis revealed that P‐type adenosine triphosphatase (ATP7B), a key gene involved in the process, was down‐regulated by CuNPs, further enhancing the sensitivity of *KRAS(G12C)* mutant LUAD to AMG‐510. The proposed cuproptosis‐oriented inducer based on copper‐containing nanoparticles presents a promising curative effect for *KRAS(G12C)* mutant LUAD, suggesting that nanomaterial‐induced cuproptosis can enhance the effectiveness of drug resistance‐related diseases, exhibiting synergistic antitumor effects against tumors.

## 2 Results

### 2.1 KRAS Mutated LUAD Patients Exhibiting Worse Survival Rate

At Shanghai Pulmonary Hospital, information was collected from 3251 LUAD patients, with an ethical approval number of K22‐318. Following patient selection (Figure 1A), survival analyses were conducted using data from 2988 patients. Notably, LUAD patients with *KRAS(G12C)* mutation exhibited a lower 5‐year survival rate compared to those with wild‐type *KRAS* (87% vs. 94%, *p* < 0.01, Figure 1B). Furthermore, in our cohort, *KRAS(G12C)* mutation emerged as an independent prognostic risk factor (Figure 1C), a finding corroborated by an additional survival analysis based on The Cancer Genome Atlas Program (TCGA) database (Figure 1D). These findings highlight the urgent need for novel therapeutic strategies. Accordingly, we propose the development of copper‐based nanomaterials capable of inducing cuproptosis in *KRAS‐mutated* LUAD, or their combination with targeted agents to further suppress *KRAS* mutant tumor cells.

**FIGURE 1:**
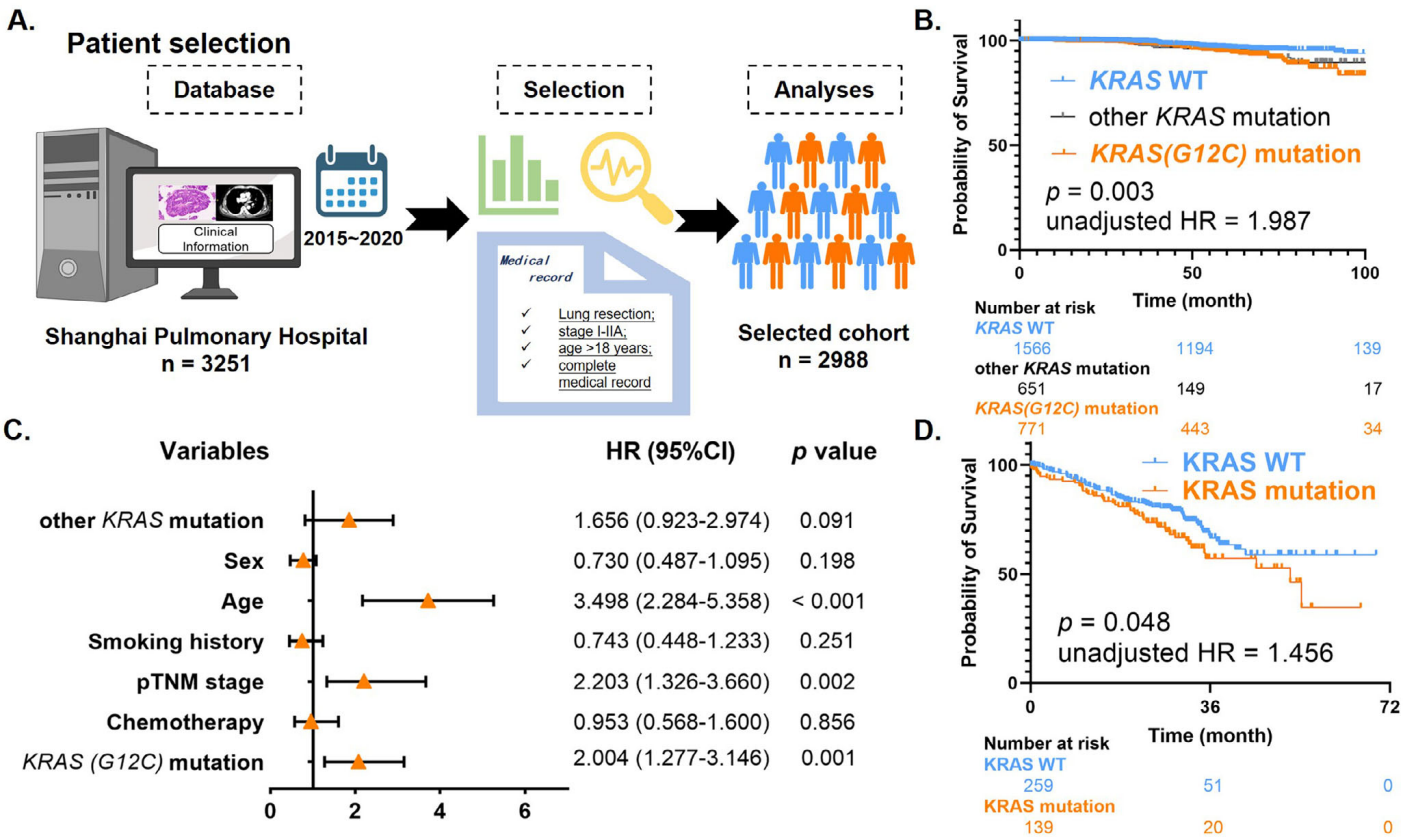
The survival of lung adenocarcinoma patients. (A) The selection process of patients. (B) Kaplan–Meier and (C) multivariable Cox regression analyses of patients from Shanghai Pulmonary Hospital. (D) Kaplan–Meier analysis of patients from TCGA database. Log‐rank test was conducted: ^***^ *p* < 0.001, ^**^ *p* < 0.01, ^*^ *p* < 0.05.

### 2.2 Characterizations of CuNPs

Copper‐based nanoparticles (CuNPs) were synthesized by encapsulating copper ions with bovine serum albumin (BSA, fraction V) under weakly alkaline pH. Transmission electron microscopy (TEM) images revealed the nanoparticulate structures of the CuNPs (Figure 2A). TEM mapping proved the existence of Cu ions in CuNPs with uniform distribution (Figure 2B). X‐ray photoelectron spectroscopy (XPS) further confirmed the presence of copper ions within the CuNPs (Figure 2C). The Cu^2+^ peak obviously existed in X‐ray Auger spectra of Cu LMM (Figure 2D), confirming the presence of Cu^2+^ rather than Cu^+^ in CuNPs [37]. The Fourier transform infrared (FT‐IR) spectrum of the CuNPs displayed amide C═O stretching and N—H bending peaks, attributed to BSA (Figure 2E). Dynamic light scattering (DLS) data showed that the hydrodynamic diameter of the CuNPs was approximately 150.8 nm in ethanol (Figure 2F). The relatively large hydrodynamic size suggests that CuNPs may form aggregates of BSA and Cu^2^^+^ under weakly alkaline conditions. Physiological stability of CuNPs was also detected by DLS. Non‐significant difference was detected in terms of hydrodynamic size at 1‐, 7‐, and 14‐day periods in phosphate buffer solution (PBS) and fetal bovine serum (FBS), respectively (Figure S1), which suggested that CuNPs could remain stable in physiological solutions. In addition, the zeta potential of CuNPs was about +8.4 mV. X‐ray diffraction (XRD) analysis indicated that the CuNPs lacked a crystalline structure (Figure 2G). Thermogravimetric analysis (TGA) indicated a final residual mass of 1.24% copper oxide, equivalent to 1% copper (Figure 2H). The copper ion release capacity of the CuNPs was significantly higher under acidic conditions compared to neutral conditions (Figure 2I). Due to the copper carbonate precipitation principle used in nanostructure, H^+^ ions in an acidic environment interact with CuCO_3_ in CuNPs, thereby producing copper ions and carbonic acid.

**FIGURE 2:**
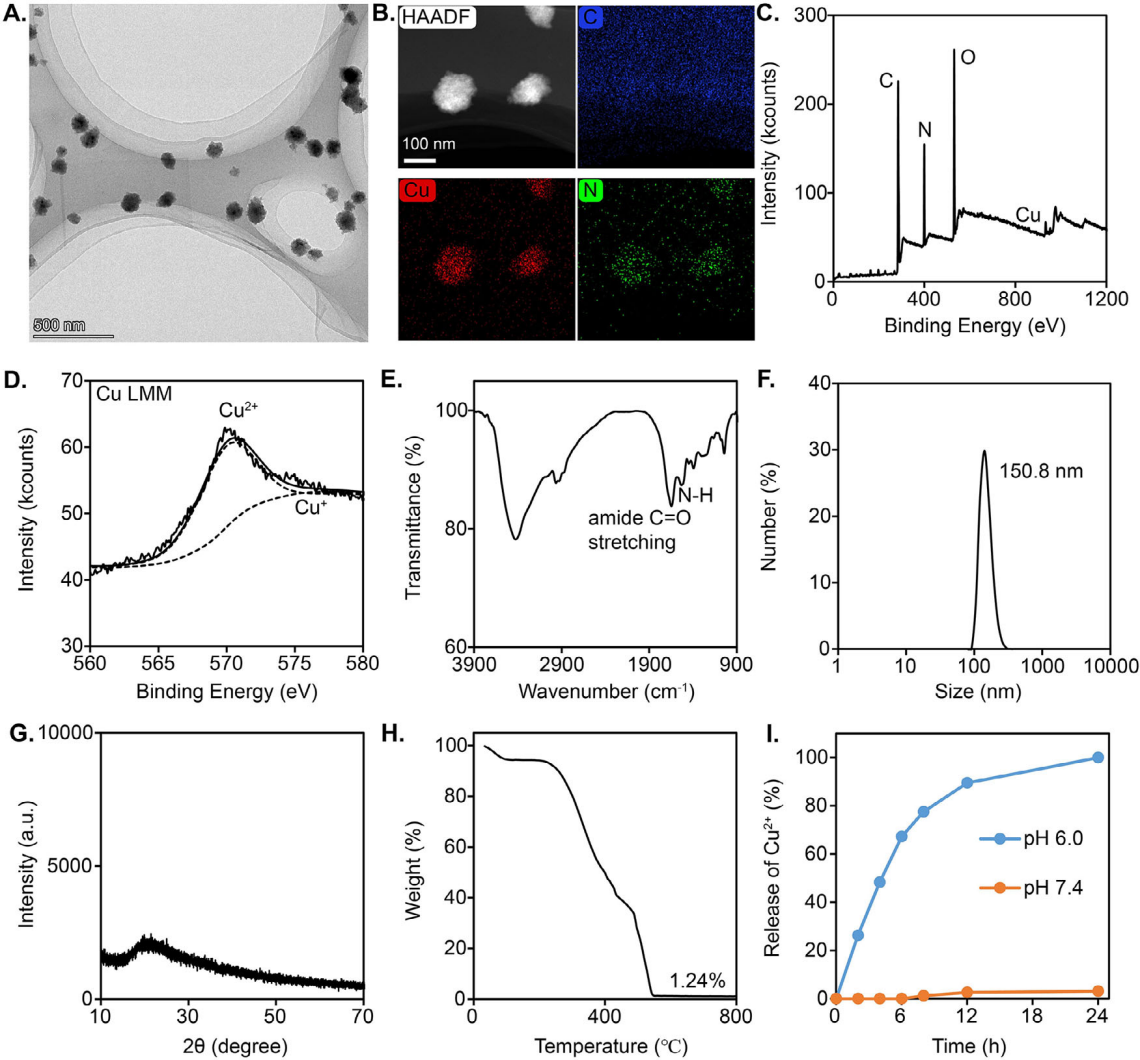
Characterizations of CuNPs. (A) TEM image of CuNPs. (B) Element mapping of CuNPs. (C) The XPS data of CuNPs. (D) XPS Auger spectra of Cu LMM of CuNPs. (E) FT‐IR analysis of CuNPs. (F) DLS data of CuNPs. (G) XRD analysis of X‐ray for CuNPs. (H) Thermogravimetric analysis of CuNPs. (I) The copper ion release capacity of CuNPs.

### 2.3 CuNPs Inhibiting Proliferation and Metastasis of LUAD Cell Lines In Vitro

The in vitro antitumor efficacy of CuNPs was evaluated using human *KRAS(G12C)*‐mutant LUAD cells (SW‐1573 and NCI‐H23). Cell counting kit‐8 (CCK8) and half‐maximal inhibitory concentration (IC50) assays revealed that CuNPs significantly inhibited the cellular activity of SW‐1573 and NCI‐H23 cell lines within 72 h, with IC50 values of 3.806 and 3.66 ppm, respectively (Figure 3A–C). Additionally, the clone formation assay produced similar results, demonstrating that CuNPs effectively hindered the growth of SW‐1573 and NCI‐H23 cells (Figure 3D,E). Flow cytometry results indicated that the apoptotic effect of CuNPs increased with concentration in SW‐1573 (Figure 3F) and NCI‐H23 (Figure 3G) cell lines. Furthermore, cell cycle experiments showed that CuNPs induced an accumulation of SW‐1573 (Figure S2) and NCI‐H23 (Figure S3) cells in the G2/M phase, suggesting that CuNPs interfere with cell cycle progression. Transwell assay results demonstrated that CuNPs significantly inhibited the migration ability of tumor cells in SW‐1573 and NCI‐H23 cells (Figure 3H,I), suggesting that CuNPs could inhibit the metastasis of LUAD cells.

**FIGURE 3:**
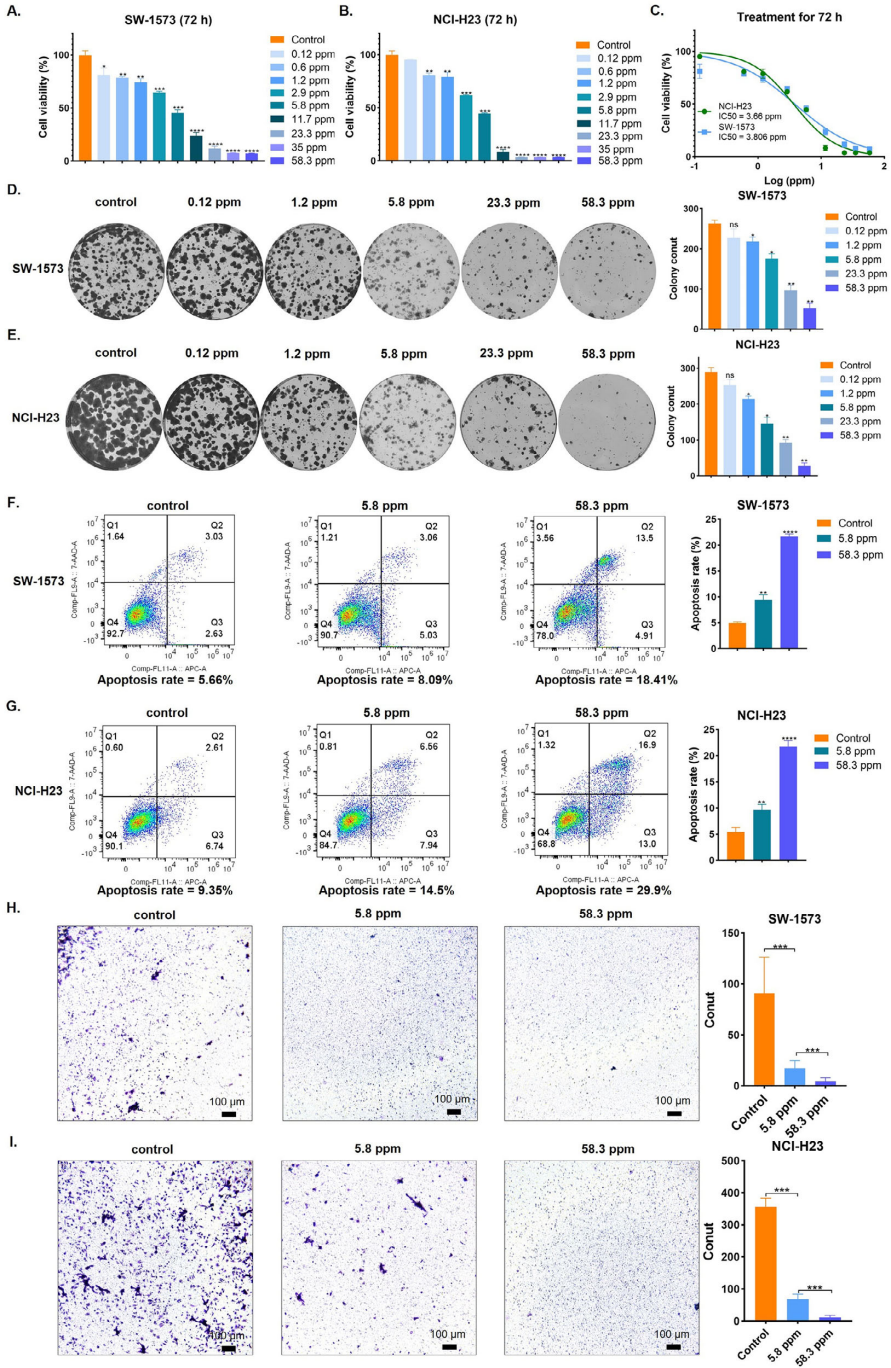
CuNPs inhibiting proliferation and metastasis of lung adenocarcinoma cell lines. Cell Counting Kit‐8 (CCK8) assays for (A) SW‐1573 and (B) NCI‐H23 after 72 h. (C) The half‐maximal inhibitory concentration assays with 72 h of CuNP treatment. A 14‐day plate cloning assay was conducted, in which 2000 cells were inoculated in each well. Subsequently, different concentrations of CuNPs were added to observe their effect on the clone formation ability of (D) SW‐1573 and (E) NCI‐H23 cells. The apoptotic effect of different concentrations of CuNPs on (F) SW‐1573 and (G) NCI‐H23 cells was assessed after 72 h using flow cytometry. The transwell assay was conducted on (H) SW‐1573 and (I) NCI‐H23 cell lines with different concentrations of CuNPs after 24 h. One‐way analysis of variance (ANOVA) was performed: ^***^ *p* < 0.001, ^**^ *p* < 0.01, ^*^ *p* < 0.05.

### 2.4 CuNPs Inducing Cuproptosis in KRAS(G12C)‐Mutated LUAD

To verify whether CuNPs induce cuproptosis in *KRAS(G12C)*‐mutant LUAD cells, SW‐1573 and NCI‐H23 cells were subjected to RNA‐seq following treatment with CuNPs. Approximately 47 genes previously identified in the literature as being associated with cuproptosis were labeled [20], and significant differences in the expression of those genes were observed in our study: upregulated and downregulated genes were consistent with this literature report (Figures S4 and S5). Using Venn diagrams, 19 and 24 cuproptosis‐related genes were found to be differentially expressed in the two cell lines, respectively (Figure 4A). Further analysis of the differentially expressed genes (DEGs) associated with cuproptosis revealed that in both SW‐1573 and NCI‐H23 cell lines (Figure 4B,C), the DEGs were primarily enriched in the lipoic acid metabolism pathway (LA pathway) following CuNPs treatment. Moreover, quantitative analysis revealed a significant downregulation (*p* < 0.01) of *ATPase copper transporting beta* (*ATP7B*), the pivotal regulator of cellular copper homeostasis. As previously reported in the literature, the expression of the LA pathway and *ATP7B* primarily serves to inhibit the onset of cuproptosis. Therefore, qPCR validation was performed for *ATP7B* and genes in the LA pathway, including lipoic acid synthetase (*LIAS*), lipoyltransferase 1 (*LIPT1*), lipoyltransferase 2 (*LIPT2*), dihydrolipoamide dehydrogenase (DLD), acyl‐CoA synthetase medium chain family member 1 (*ACSM1*), and ferredoxin 1 (*FDX1*). As shown in Table S1 and Figure 4D,E, CuNPs significantly inhibited the expression of *ATP7B*, *LIAS*, *LIPT1*, *LIPT2*, *ACSM1*, *DLD*, and *FDX1* in both SW‐1573 and NCI‐H23 cell lines, although the suppression of *DLD* and *ACSM1* was relatively modest. In Western blotting, the expression of LIAS gradually decreased with increasing doses of CuNPs, while the corresponding oligomerization of lipoylated dihydrolipoamide S‐acetyltransferase (DLAT) progressively increased in SW‐1573 and NCI‐H23 cells (Figure 4F–H). Immunofluorescence results showed that the oligomerization of DLAT became more pronounced with higher concentrations of CuNPs (Figure 4I,J). The presence of DLAT oligomerization indicated that CuNPs can indeed induce cuproptosis in *KRAS(G12C)*‐mutated LUAD cells.

**FIGURE 4:**
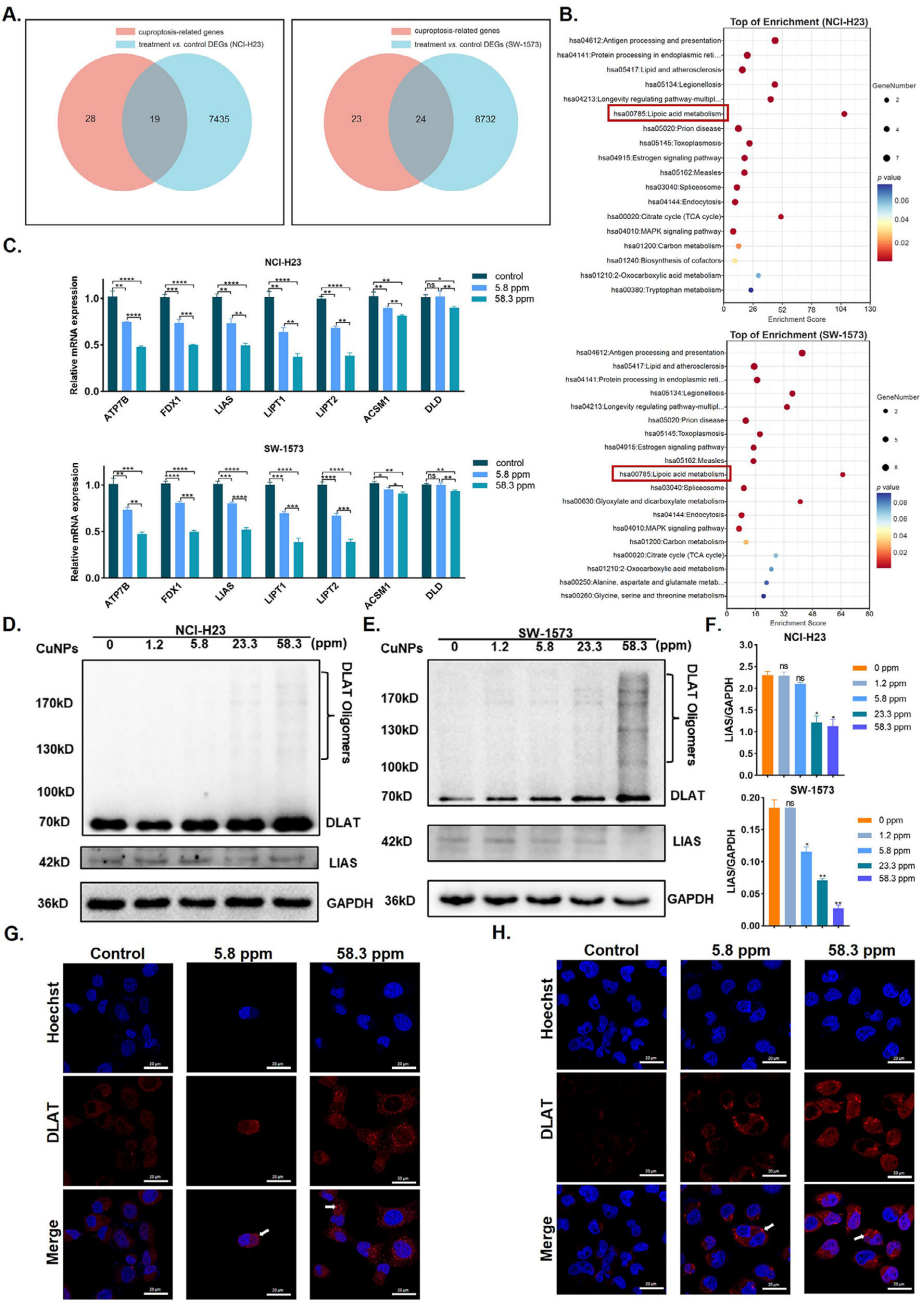
CuNPs inducing cuproptosis in *KRAS(G12C)*‐mutated lung adenocarcinoma. SW‐1573 and NCI‐H23 cells are subjected to RNA‐seq after 48 h stimulation with PBS and 10 times IC50 concentrations of CuNPs (three replicates per group), respectively. (A) Venn diagrams of DEGs and cuproptosis‐related genes. (B) KEGG analyses of intersecting cuproptosis‐related genes in NCI‐H23 and (C) SW‐1573 cells. (D) qPCR validation of *ATP7B* and LA pathway in NCI‐H23 and (E) SW‐1573 cells. (F) Western blotting for cuproptosis‐related protein (DLAT oligomers and LIAS) in NCI‐H23 and (G) SW‐1573 cells. (H) The corresponding western‐blotting semiquantitative data. (I) DLAT aggregation fluorescence images of NCI‐H23 and (J) SW‐1573 cells with different treatments (PBS, 5.8 ppm, 58.8 ppm CuNPs) after 72 h. White arrows indicated DLAT aggregation. Scale bars: 20 µm. Student's *t*‐test was performed: ^*^ *p* < 0.05, ^**^ *p* < 0.01, ^***^ *p* < 0.001.

Beyond these cuproptosis‐related changes, CuNPs also elevated intracellular reactive oxygen species (ROS) levels and altered the redox microenvironment (Figure S6), prompting further validation to exclude other cell death modalities. Validation experiments for other cell death modalities were performed. Ferrostatin‐1 (2 µM) was used to determine whether CuNPs could induce ferroptosis in LUAD cells, with RSL3 serving as a positive control. As shown in Figure S7, experimental results indicated that ferrostatin‐1 could not completely block ferroptosis. Additionally, three apoptosis inhibitors (10 µM) were used to determine the percentage of apoptosis in LUAD cells caused by CuNPs, with cisplatin (1 µM) as a positive control. The proportion of apoptosis blocked by necrosulfonamide, Z‐VAD‐FMK, and necrostatin‐2 racemate was much smaller than that blocked by cisplatin (Figure S8). This further suggests that tumor cells mainly underwent cuproptosis following CuNPs treatment.

### 2.5 CuNPs Improving AMG‐510 Sensitivity in KRAS(G12C)‐Mutated LUAD

The therapeutic effect of traditional chemotherapy on patients of *KRAS(G12C)* mutated LUAD is limited [38, 39]. Inhibitors such as AMG‐510, which have been marketed in recent years, are still not effective enough for single‐agent use to further meet the need for survival benefits for those patients [19, 40, 41]. Thus, there is a need to develop new antitumor agents or find means to sensitize AMG‐510 in this cohort. Regarding the *KRAS(G12C)*‐mutated LUAD cell lines, although they share the same KRAS mutation site, different cell lines exhibit varying responses to AMG‐510. Sensitivity experiments conducted on three *KRAS(G12C)*‐mutated LUAD cell lines revealed that the 72‐h IC50 values for the NCI‐H23 and SW‐1573 cell lines were 90‐fold and 200‐fold higher than that of the NCI‐H358 cell line, respectively. A previous study reported similar results [42]. This indicates that the NCI‐H23 and SW‐1573 cell lines are significantly less sensitive to AMG‐510 compared to the NCI‐H358 cell line (Figure 5A–C and Figures S9 and S10). Based on these results, NCI‐H358 was selected as AMG‐510 treatment‐sensitive cells, while NCI‐H23 and SW‐1573 were used as AMG‐510 treatment‐insensitive or naturally drug‐resistant cells. Importantly, our findings further demonstrated that CuNPs can induce cuproptosis in LUAD cell lines, providing a new perspective for the treatment of LUAD.

**FIGURE 5:**
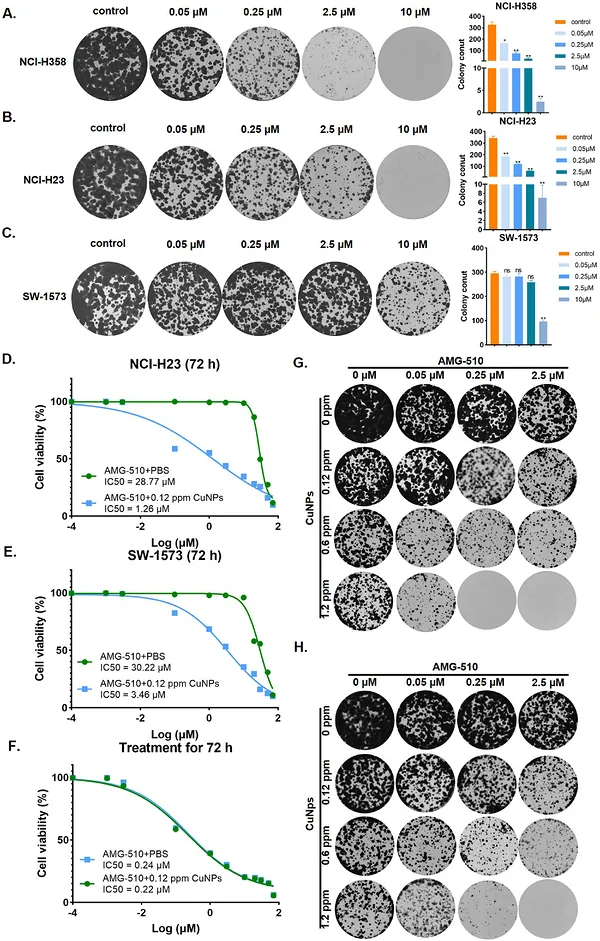
CuNPs improve AMG‐510 sensitivity in *KRAS(G12C)*‐mutated LUAD in vitro. A 14‐day plate cloning assay was conducted, where 2000 cells were inoculated in each well. Subsequently, different concentrations of AMG‐510 were added to observe their effect on the clone formation ability of (A) NCI‐H358, (B) NCI‐H23, and (C) SW‐1573 cells. The half‐maximal inhibitory concentration assays with 72 h of the combination of AMG‐510 and 0.12 ppm CuNPs treatment in (D) NCI‐H23, (E) SW‐1573, and (F) NCI‐H358 cell lines. (G) A 14‐day plate cloning assay for NCI‐H23 and (H) SW‐1573 cell lines after treatment of AMG‐510 and CuNPs. Student's *t*‐test was performed: ^*^ *p* < 0.05, ^**^ *p* < 0.01, ^***^ *p* < 0.001.

The NCI‐H23 and SW‐1573 cell lines used in this experiment are both AMG‐510‐insensitive, but they responded well to CuNPs. Therefore, the combination of AMG‐510 and CuNPs was applied to explore whether CuNPs could sensitize AMG‐510. A significant reduction in the 72‐h IC50 of AMG‐510 was observed in the SW‐1573 and NCI‐H23 cell lines (*p* < 0.05), whereas the sensitization was not significant in the NCI‐H358 cell line (Figure 5D–F). The combination index (CI) of drugs was smaller than 1 in the NCI‐H23 and SW‐1573 cell lines (Tables S2 and S3), which indicated the synergistic antitumor effect in the combination of CuNPs and AMG‐510. In the two AMG‐510‐insensitive NCI‐H23 and SW‐1573 cell lines, the effect of the combination of CuNPs and AMG‐510 was further demonstrated through orthogonal experiments (Figures S11 and S12) and plate cloning experiments (Figure 5G,H). To further explore whether other copper‐based materials share similar biological effects, we referred to a previous study showing that Cu‐MOF can induce both apoptosis and cuproptosis in tumor cells [25]. Based on these findings, we investigated whether Cu‐MOF could enhance the anti‐tumor efficacy of AMG‐510. Our results revealed that Cu‐MOF indeed sensitized tumor cells to AMG‐510, whereas the addition of copper chloride solution alone failed to potentiate the anti‐tumor effects of AMG‐510 (Figure S13). These experiments confirmed that CuNPs could sensitize AMG‐510, potentially overcoming treatment resistance to AMG‐510.

### 2.6 ATP7B Is a Potential Target for AMG‐510 Resistance

Based on the aforementioned results, CuNPs were confirmed to induce cuproptosis and sensitize AMG‐510 in *KRAS(G12C)*‐mutated LUAD cells. The potential association between cuproptosis and drug sensitization needs to be further explored. The up‐regulated DEGs in NCI‐H23 versus NCI‐H358 and SW‐1573 versus NCI‐H358 were screened out, with the number of DEGs being 3215 and 2969, respectively, which might be related to AMG‐510 insensitivity or resistance. The down‐regulated DEGs in NCI‐H23 and SW‐1573 treated with CuNPs were screened out, with the numbers being 3574 and 3854, respectively, which were genes related to the action of CuNPs. An integration analysis of the up‐regulated AMG‐510‐related genes and the down‐regulated CuNPs‐related genes was performed, resulting in 204 DEGs as shown in Figure 6A. Finally, these 204 DEGs were intersected with the set of genes related to cuproptosis, and only *ATP7B* was identified (Figure 6A). Further RNA‐seq analysis found that the expression of *ATP7B* in the two AMG‐510‐insensitive cell lines was significantly higher than in the AMG‐510‐sensitive cell lines (Figure 6B). In contrast, the expression of *ATP7B* significantly decreased after CuNPs treatment (Figure 6C,D). Consistent with our findings, re‐analysis of published genome‐wide CRISPR‐Cas9 screening data on another KRAS(G12C) inhibitor (ARS‐853) also revealed a significant upregulation of *ATP7B* following pharmacological inhibition of KRAS(G12C) (Figure S14) [43], further supporting *ATP7B* as a potential resistance target. This phenomenon implies that *ATP7B* may be a resistance target for KRAS(G12C) inhibitors.

**FIGURE 6:**
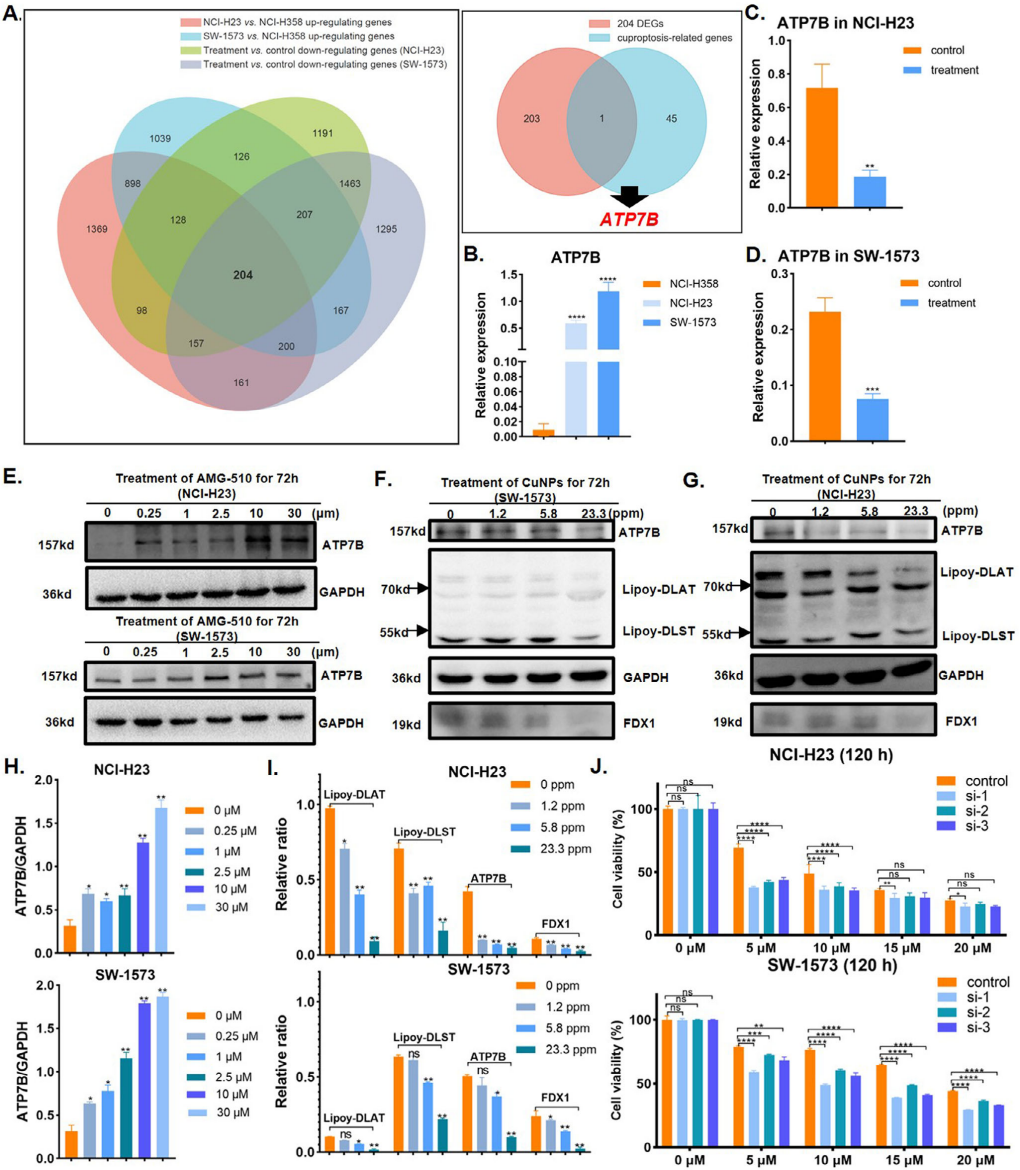
*ATP7B* may be a target for AMG‐510 resistance. Three cell lines were subjected to RNA‐seq without drug treatment, and both SW‐1573 and NCI‐H23 were treated with 5‐fold IC50 CuNPs for 24 h. (A) An integration analysis for screening *ATP7B*. (B) The *ATP7B* expression in the data of RNA‐seq. RNAseq uncovered the *ATP7B* expression in (C) NCI‐H23 and (D) SW‐1573 cell lines after the treatment of CuNPs. (E) Western blot uncovered the ATP7B level with the treatment of AMG‐510 after 72 h. The results of western blotting in (F) NCI‐H23 and (G) SW‐1573 cell lines with the treatment of CuNPs after 72 h. The corresponding western‐blotting semiquantitative data of (H) AMG‐510 and (I) CuNPs treatment. (J) Cell viability of NCI‐H23 and SW‐1573 cell lines with *siATP7B* after treatment of AMG‐510. Student's *t*‐test was performed: ^*^ *p* < 0.05, ^**^ *p* < 0.01, ^***^ *p* < 0.001.

The expression level of ATP7B in the three cell lines was verified by western blot. As expected, the lowest level of ATP7B expression was found in the NCI‐H358 cell line (Figure S15). Subsequent induction of the cell lines with AMG‐510 revealed that the expression level of ATP7B protein increased with the increasing dose of AMG‐510 (Figure 6E), suggesting a potential correlation between ATP7B expression and AMG‐510 sensitivity. However, the expression level of ATP7B decreased with increasing doses of CuNPs treatment (Figure 6F,G). The bands of cuproptosis‐related proteins, such as FDX1, lipoylated DLAT (Lipoy‐DLAT), and lipoylated dihydrolipoamide S‐succinyltransferase (Lipoy‐DLST), also decreased, consistent with the trend of cuproptosis, suggesting that CuNPs could decrease ATP7B protein levels. The corresponding western blotting semiquantitative data are shown in Figure 6H,I.

Then, we knocked down the *ATP7B* gene with three siRNAs in two AMG‐510‐insensitive cell lines and found that, at the same concentration of AMG‐510, the siRNA knockdown cell lines had lower cell survival (Figure 6J). Additionally, the AMG‐510‐sensitive NCI‐H358 cell lines were treated by overexpressing ATP7B, and it was found that the IC50 of AMG‐510 increased 7‐fold in the ATP7B overexpressing cell lines (Figures S16 and S17; Figure 6J). These results suggest that *ATP7B* is a potential resistance target of AMG‐510. While *ATP7B* has previously been implicated in cisplatin resistance [44, 45, 46], our study is the first to demonstrate its association with resistance to a targeted KRAS(G12C) inhibitor, highlighting a novel role for this cuproptosis‐related protein. To explore the role of *ATP7B* in the regulatory network of targeted drug resistance, RNA‐seq data and TCGA data of cell lines were used for pathway and cluster analysis. Additionally, published RNA‐seq data from *ATP7B* knockout and wild‐type cell lines were utilized in this study [47]. KEGG analysis suggested that the DEGs were enriched in the calcium signaling pathway. The genes involved in the calcium signaling pathway were further clustered and analyzed, with results shown in Figure S18.

### 2.7 CuNPs and AMG‐510 in Animal Experiments

In vitro experiments have demonstrated the ability of CuNPs to induce cuproptosis and sensitize targeted drugs. Therefore, their safety and efficacy in vivo need to be further evaluated in preparation for subsequent clinical applications. As shown in Figure 7A, for in vivo experiments, mice with measurable tumor volumes were randomly divided into four groups: the control group (*n* = 7), the CuNPs group (*n* = 7), the AMG‐510 group (*n* = 7), and the combination group (*n* = 6). As shown in Figure 7B–D, the tumors of the mice in the combination group were the smallest among the four groups (all *p* < 0.05). In the tumor growth curves, the tumors in the drug‐alone groups only showed a weakened growth trend but were still in the growth stage, while the tumor growth trend in the combination group was essentially interrupted (Figure 7E). The tumor inhibition rate reached 91% in the combination group, markedly higher than AMG‐510 (46%) or CuNPs alone (56%), consistent with clinical observations that AMG‐510 monotherapy yields limited response rates [16, 40, 41]. Pathological analyses further supported these findings: Ki‐67 staining showed the weakest proliferative activity, and TUNEL staining revealed the highest apoptotic index in the combination group (Figure 7F,G). Importantly, the combination treatment did not cause weight loss or organ damage in mice (Figure S19), indicating favorable safety in vivo.

**FIGURE 7:**
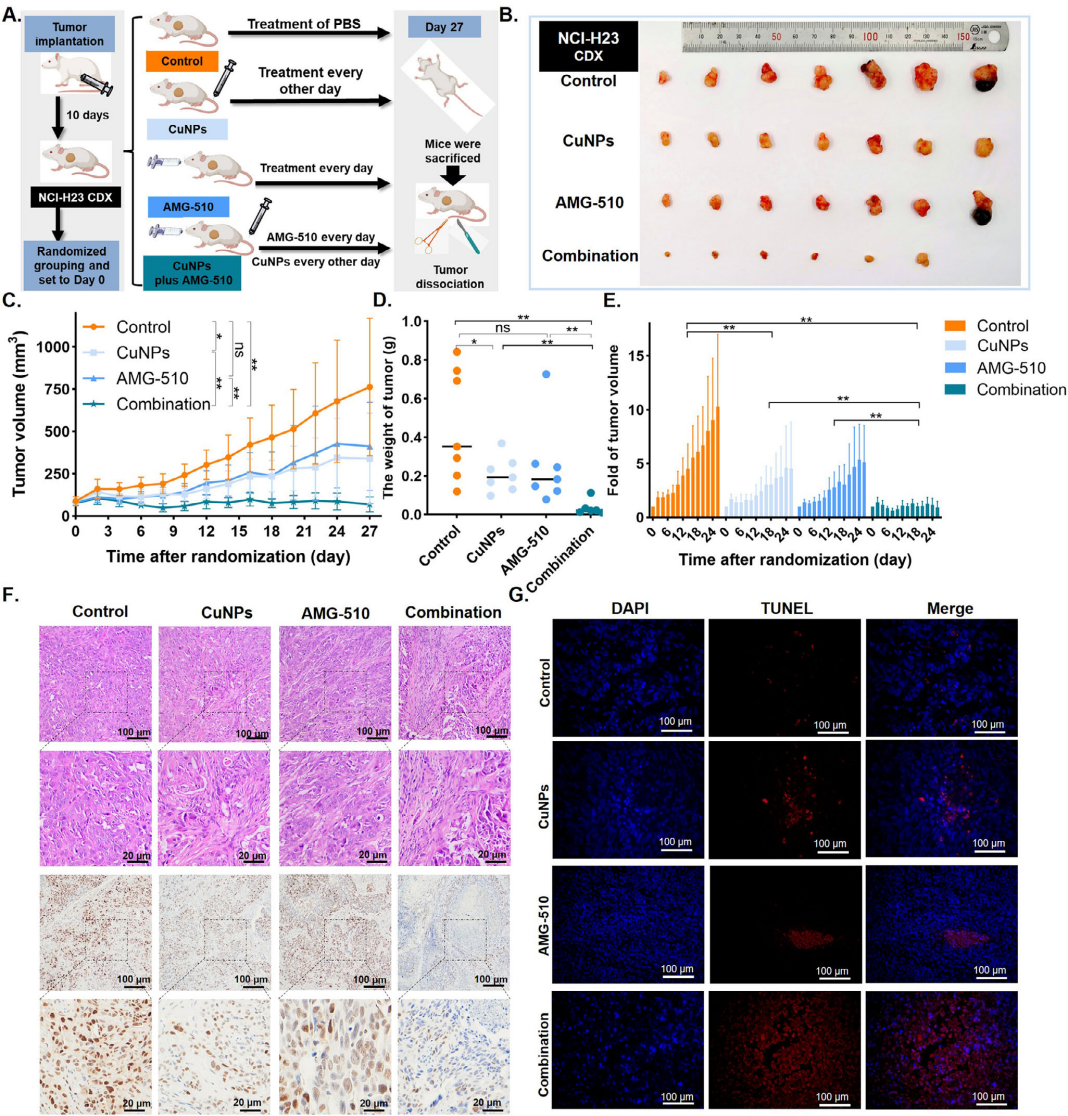
CuNPs and AMG‐510 in animal experiments. (A) The flowchart of animal experiments (control: *n* = 7; CuNPs: *n* = 7; AMG‐510: *n* = 7; combination: *n* = 6). (B) Photographs of tumors after treatments with NS, 30 mg/kg AMG‐510, 100 µg CuNPs per mouse, and 30 mg/kg AMG‐510 plus 100 µg CuNPs. (C) Tumor growth curves during the 28‐day therapeutic period. (D) The tumor weight on Day 27. (E) Tumor multiplication curves during the 28‐day therapeutic period. (F) Hematoxylin‐eosin staining and Ki‐67 staining of tumor tissue. (G) TUNEL staining. Student's *t*‐test was performed: ^*^ *p* < 0.05, ^**^ *p* < 0.01, ^***^ *p* < 0.001.

## 3 Discussion

In this study, we developed CuNPs composed of bovine serum albumin encapsulating copper ions, which demonstrated potent anti‐tumor activity by inducing cuproptosis in LUAD cells. These CuNPs effectively suppressed LUAD proliferation and metastasis, and notably enhanced the sensitivity of *KRAS(G12C)*‐mutated LUAD to the targeted therapy AMG‐510. Through mechanistic investigation, we identified that *ATP7B*‐a key gene involved in copper transport and cuproptosis regulation‐ as a contributor to AMG‐510 resistance, potentially via modulation of the calcium signaling pathway (Figure S18). *ATP7B* thus emerges as a promising target or biomarker for overcoming resistance and improving drug responsiveness (Scheme 1). The transcriptomic data further verified that *ATP7B* was down‐regulated by the CuNPs‐induced cuproptosis (Figure 6A). This study is the first to demonstrate that CuNPs‐mediated cuproptosis can effectively reverse drug resistance in LUAD when combined with AMG‐510. Although research on KRAS(G12C) inhibitors remains in its early stages, current clinical data suggest that their therapeutic efficacy is still insufficient to meet clinical needs [19, 40, 41]. The development of these inhibitors may have addressed the scientific challenge of targeting a previously “undruggable” protein, but significant hurdles remain in translating this progress into meaningful clinical outcomes. Now that cuproptosis‐related drugs such as disulfiram have entered clinical trials [48, 49, 50], the application of this novel cell death pathway in LUAD therapy holds great promise. We believe that the proposed strategy of interfering with cellular cuproptosis will provide new therapeutic approaches for lung cancer treatment.

**SCHEME 1:**
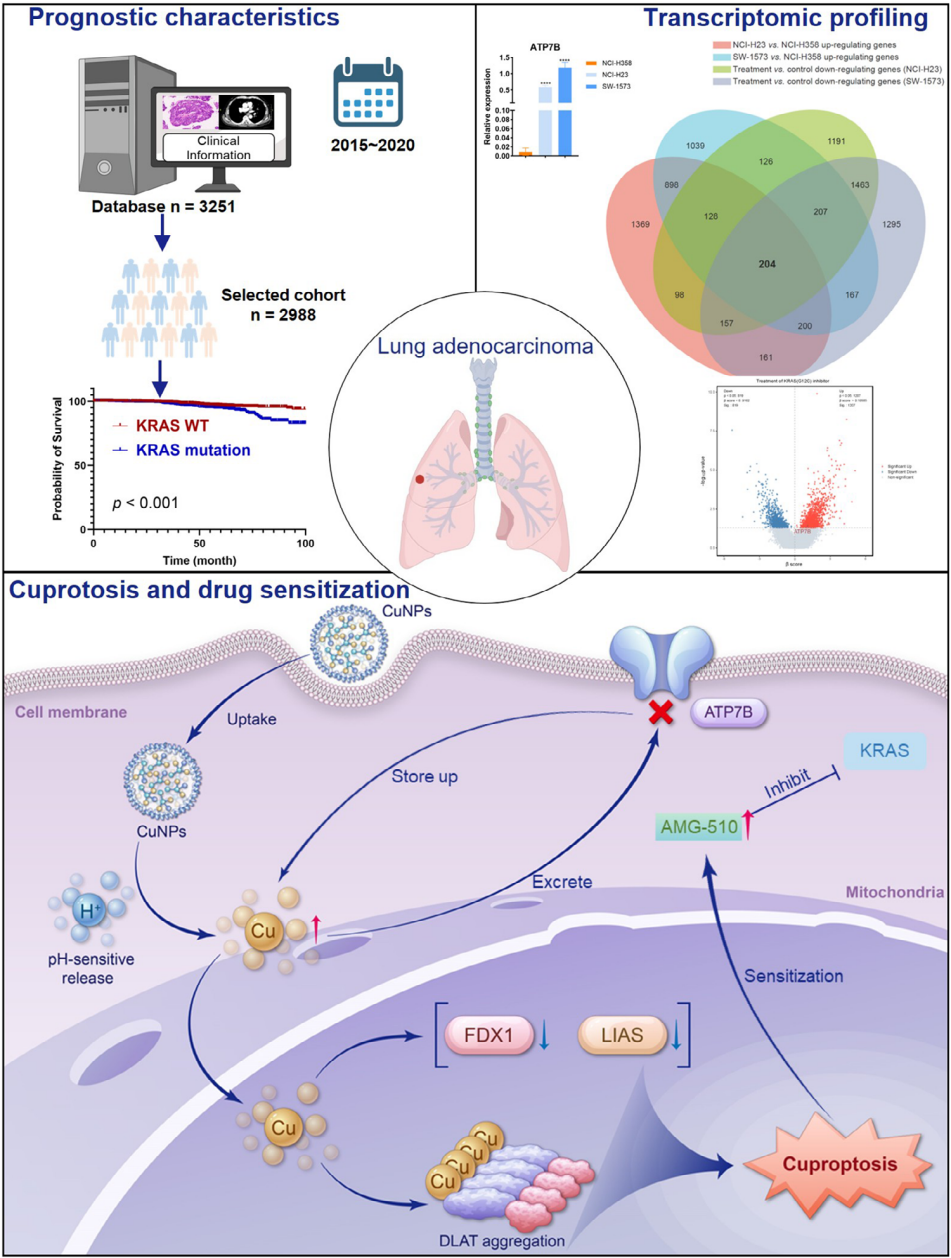
The schematic illustration of cuproptosis induced by CuNPs. By analyzing patient data, we found that KRAS‐mutated LUAD has a worse survival rate compared to wild‐type LUAD. In comparing cell lines with varying sensitivities to AMG‐510, we observed that *ATP7B*—a key regulator of cuproptosis—was up‐regulated in AMG‐510‐resistant cell lines, increasing their sensitivity to cuproptosis. The CuNPs released copper ions in an acidic environment to trigger cuproptosis for the overcoming of AMG‐510 resistance in a synergistic antitumor effect.

In our study, SW‐1573 and NCI‐H23 were identified as cell lines exhibiting insensitivity to AMG‐510 (Figure 5). This finding is consistent with previous research, which also reported that NCI‐H23 is unresponsive to AMG‐510 [42]. However, the sensitivity of SW‐1573 to AMG‐510 remains insufficiently characterized and warrants further investigation. Notably, earlier studies have shown that both SW‐1573 and NCI‐H23 are resistant to another KRAS(G12C)‐targeted inhibitor, ARS‐1620 [51]. Based on our experimental data, we propose that the responsiveness of SW‐1573 and NCI‐H23 to AMG‐510 is indeed lower than that of NCI‐H358. Nevertheless, this observation requires further validation through independent studies to confirm its reproducibility and underlying mechanisms.

Our prior investigations, leveraging publicly available databases from TCGA and GEO, demonstrated the significant prognostic value of cuproptosis‐related genes in LUAD, particularly in predicting patient outcomes and therapeutic responsiveness [26]. These findings underscore the pivotal role of cuproptosis in LUAD pathogenesis and highlight its potential as a promising target for therapeutic intervention. Motivated by these insights, we developed CuNPs through the conjugation of copper ions with BSA. Physical and chemical data showed CuNPs are prone to degradation under weak acidic conditions in the present study, thereby releasing copper ions to induce cuproptosis for the treatment of LUAD (Figure 2). The acidic nature of the tumor microenvironment enables selective degradation of CuNPs, thereby conferring inherent tumor‐targeting properties. To assess the physiological stability of CuNPs following intravenous administration in mice, we conducted zeta potential measurements at multiple time points (Figure S1). The results demonstrated that the nanoparticles maintained consistent surface charge characteristics over time, indicating their relative stability under physiological conditions.

Building upon our demonstration that CuNPs effectively inhibit the metastasis and progression of LUAD (Figure 2), we investigated the combinatorial anti‐tumor effects of CuNPs with targeted therapeutic agents (Figure 5). Our findings revealed a synergistic lethal interaction between CuNPs and AMG‐510, indicating enhanced therapeutic efficacy when used in combination. Subsequent bioinformatic analysis identified *ATP7B* as a potential mediator of drug resistance (Figure 6). Literature‐based evidence indicates that *ATP7B* modulates calcium ion signaling pathways, in which EGFR and CALM3 (Calmodulin 3) are known to play critical roles in mediating resistance to chemotherapy and targeted therapies [52, 53]. Therefore, *ATP7B* may contribute to targeted drug resistance by regulating these genes within the calcium signaling pathway (Figure S18). These findings collectively position ATP7B and its associated regulatory network as promising targets for overcoming drug resistance in cancer treatment.

When cuproptosis was first discovered and its underlying mechanisms explored, in vitro studies typically involved the addition of 2 µM copper ions to the culture medium, along with copper ionophores to facilitate the transport of extracellular copper into cells, thereby inducing cuproptosis [21]. However, under physiological conditions, a copper ion concentration of 2 µM outside the cell could easily cause toxic reactions. Currently, there is no suitable drug that can specifically target and increase copper ion content in tumor cells. The designed CuNPs not only release copper ions in response to the acidic tumor environment but have also demonstrated their safety in vivo (Figure S19). As a monotherapy, CuNPs showed significant antitumor efficacy. Moreover, when combined with AMG‐510, their cancer‐inhibitory effects were markedly enhanced, highlighting the therapeutic potential of this synergistic approach.

Emerging evidence indicates that numerous malignancies can be targeted by pharmacologically inducing cuproptosis using specific drugs or compounds. Currently, clinical applications of copper ionophores—such as Elesclomol—require co‐administration with copper salt solutions to initiate cuproptosis in tumor cells. However, systemic delivery of these agents lacks tumor‐specific targeting, raising concerns regarding their safety. In contrast, the synthesized CuNPs exhibit a favorable safety profile in preclinical in vivo studies while demonstrating efficacy comparable to that of targeted therapies (Figure 7). Accumulating research suggests that targeted induction of cuproptosis not only directly eliminates tumor cells but also enhances antitumor immune responses [34]. Consequently, the CuNPs hold significant translational potential, particularly in combination with immunotherapy for localized treatment of superficial tumors—such as peritumoral injections in breast cancer, head and neck squamous cell carcinoma, and melanoma. Additionally, the CuNPs may offer therapeutic benefits for malignancies lacking targeted treatment options, including esophageal squamous cell carcinoma and small cell lung cancer. Further preclinical investigations are warranted to validate these applications and optimize the clinical utility of CuNP‐based cuproptosis induction strategies.

## 4 Conclusions

In summary, we developed BSA‐encapsulated copper nanoparticles (CuNPs) to overcome AMG‐510 resistance in LUAD treatment. Our comprehensive in vitro and in vivo studies demonstrated that CuNPs effectively induce cuproptosis and exhibit significant anti‐tumor activity against LUAD. Mechanistically, CuNPs were shown to potentiate AMG‐510's therapeutic efficacy through targeted inhibition of *ATP7B*, a critical regulator of cuproptosis. These findings not only provide important insights into overcoming AMG‐510 resistance but also highlight the therapeutic potential of cuproptosis induction in cancer treatment.

## Author Contributions

**Leilei Wu**: conceptualization, methodology, visualization, writing – original draft, writing – review and editing. **Kun Li**: conceptualization, investigation, writing – review and editing. **Han Wang**: conceptualization, methodology, investigation, visualization, writing – original draft, writing – review and editing. **Shangshang Ma**: methodology, investigation, writing – review and editing. **Rangrang Wang**: methodology, investigation, writing – review and editing. **Yongfei Fan**: methodology, writing – review and editing. **Xichun Qin**: methodology, writing – review and editing. **Mingjun Li**: writing – review and editing. **Diego Gonzalez‐Rivas**: investigation, writing – review and editing. **Xiuru Ji**: methodology, writing – review and editing. **Dalong Ni**: conceptualization, supervision, writing – review and editing. **Dong Xie**: conceptualization, supervision, writing – review and editing.

## Conflicts of Interest

The authors declare no conflicts of interest.

## Ethics Statement

The Clinical Research Ethics Committee of Shanghai Pulmonary Hospital approved this study, and the human data was in accordance with the Declaration of Helsinki in the manuscript, with the need for informed consent waived for this retrospective study (ethical approval number of K22‐318). All animal studies have been approved by the Review Board and Ethics Committee of Shanghai Pulmonary Hospital (ID: K22‐390).

## Supporting information




**Supporting File**: exp270250‐sup‐0001‐SuppMat.docx.
