# Supplementary material for: A Cuproptosis Nanoparticle Reverses Lung Adenocarcinoma Drug Resistance
Source: Exploration (Beijing). 2026 Nov 10:70250. Online ahead of print. doi: 10.1002/exp2.70250 (PMC13752522; doi:10.1002/exp2.70250)
Supplement: Supplementary file 1 [file EXP2-9999-0-s001.docx]

**SUPPORTING INFORMATION**

A Cuproptosis Nanoparticle Reverses Lung Adenocarcinoma Drug Resistance

**Materials and Methods**

***Preparation of CuNPs***

CuCl_2_ solution (30 mM, 3 mL, Macklin, China) and bovine serum albumin (fraction V) solution (5 mg/mL, 30 mL, Macklin, China) were mixed. Then Na_2_CO_3_ solution (450 mM, 0.3 mL, Macklin, China) and HCl solution (37%, 0.1 mL, Greagent, China) were added to this solution and further heated under 55 ℃ for 2 hours. The obtained CuNPs solution was light green. Finally, CuNPs were purified by high-speed centrifuge and washed by water for three times.

***Experimental apparatus***

TEM image was carried out by FEI Talos F200X. XRD was measured by Rigaku D/MAX-2250 V. XPS spectra were measured by Thermo Fisher Scientific ESCALAB 250XI. FTIR was carried out by Thermo Fisher Scientific Nicolet iS20. Dynamic light scattering was measured by Malvern Zetasizer Nano S. The content of Cu was measured by inductively coupled plasma optical emission spectrometry (ICP-OES, Agilent 725). TGA was detected by NETZSCH TG 209 F3.

***Cell lines, cell culture, and drug treatment***

The LUAD cell lines NCI-H23, NCI-H358, and SW-1573 were obtained from the American Type Culture Collection. The NCI-H23 and NCI-H358 cell lines were cultured in Roswell Park Memorial Institute 1640 medium (WISENT Inc., Nanjing, China) supplemented with 10% fetal bovine serum (Procell Life Science &. Technology Co., Ltd., Wuhan, China) and 50 μg/ml penicillin–streptomycin (Invitrogen). The SW-1573 cell lines were cultured in Dulbecco's Modified Eagle medium (WISENT Inc., Nanjing, China) supplemented with 10% fetal bovine serum (Procell Life Science &. Technology Co., Ltd., Wuhan, China) and 50 μg/ml penicillin-streptomycin (Invitrogen). All cells used in this study were maintained in a 5% CO_2_ cell culture incubator at 37 °C. Notably, all cell lines harbor *KRAS(G12C)* mutations. AMG-510, Ferrostatin-1, RSL3, cisplatin, Necrosulfonamide, Z-VAD-FMK, Necrostatin-2 racemate, and Neomycin sulfate were purchased from TargetMol Co. Ltd., (USA).

***Cell viability assay, IC50 determination, and colony formation assay***

To determine the IC50 values of CuNPs, AMG-510, and RSL3 in LUAD cell lines, we evaluated cell viability with a Cell Counting Kit-8 (CCK8; TargetMol Co. Ltd., USA) after drug treatment. In this assay, 2000 cells were seeded in 96-well plates, incubated for confluent growth in complete medium, and subsequently incubated with gradient concentrations of CuNPs, AMG-510, and RSL3 as single agents or as constant ratio combinations. Ferrostatin-1 (2 μM) was used to block ferroptosis. After drug treatment, we aspirated the old medium, added fresh complete medium containing 10% CCK8 reagent, and incubated the plates at 37 °C for two hours. Notably, the plates were kept in the dark throughout this process. An Infinite M Plex microplate reader (Tecan Trading Co., Ltd., Shanghai, China) and Tecan.At.XFluor software were used for absorbance acquisition at a detection wavelength of 450 nm. Dose–response curves were fitted using nonlinear regression with a four-parameter logistic model (variable slope) in GraphPad Prism software version 9.0 (GraphPad Software, San Diego, CA, USA). IC50 values were calculated from the fitted curves. For the colony formation assay, cells were seeded into 6-well plates (1000-2000 cells/well). Then, the medium containing the drug was replaced every other day. After 14 days of culture, the colonies were fixed with 4% paraformaldehyde and stained with 0.5% crystal violet (Yeasen Biotech Co., Ltd., Shanghai, China). The colonies were then counted.

***Apoptosis Detection***

Cells were incubated in 24-well plates and exposed to different concentrations of CuNPs for the indicated time. Subsequently, cells were stained with Annexin V/ propidium iodide (PI) (Elabscience, Wuhan, China). The staining was performed according to the manufacturer’s instructions (https://789.bio/ea/KiXLaH). After staining, cell apoptosis was analyzed using a BD FACSCanto II flow cytometer (BD BioScience, San Jose, CA, USA).

***Cell Cycle Analysis***

After treatment with different concentrations of MCuNPs for the indicated time, lung adenocarcinoma cells were harvested, washed with cold PBS, permeabilized using 70% ethanol overnight at −20 °C, and then incubated with 300 μL PI/RNase staining buffer (Elabscience, Wuhan, China) at room temperature for 15 min followed by flow cytometric analysis. Data was analyzed using ModFit LT software.

***Lentivirus packaging, cell transfection, and immunoblotting***

To construct *ATP7B*-overexpression cell lines, human embryonic kidney 293 packaging cells were used for lentivirus production. To generate lentiviral particles, 293T cells were transiently transfected with vector DNA along with the packaging plasmid psPAX2 (#12260, Addgene), the envelope plasmid pMD2.G (#12,259, Addgene), and the target plasmid (pcDNA3.1-ATP7B-3*Flag-Neomycin and pLVX-Flag-IRES-Puro) using Lipofectamine 3000 (Thermo Fisher Scientific, Waltham, MA, USA) according to the manufacturer’s protocol (https://tools.thermofisher.cn/content/sfs/manuals/lipofectamine3000_protocol.pdf). After 48 h of culture, the virus-containing supernatants were collected and filtered through a 0.45 μm filter. NCI-H358 cell lines were infected with a suspension of the virus-containing supernatant and incubated for 24 h at 37 °C. Stable cells were selected with 50 μg/mL Neomycin sulfate for ten days. The siRNA transfection (50 nM, www.geneppl.com)^1^ was performed using Lipofectamine 3000 in cell lines of NCI-H23 and SW-1573. For immunoblotting, cells were washed once with ice-cold PBS and lysed in lysis buffer (50 mM Tris-HCl, pH 7.4; 150 mM NaCl; 1 mM EDTA; 1% Triton X-100; 5% glycerol; and proteinase inhibitor cocktail) for 15 min at 4°C. The supernatant was separated via centrifugation for 15 min at 12,000 rpm and 4°C. Proteins were resolved on SDS‒PAGE gels and detected by an electrochemiluminescence (ECL) imaging system (Tanon). Primary antibodies specific for the following molecules were used: ATP7B (1:1000; 19786-1-AP, Proteintech), LIAS (1:1000; 11577-1-AP, Proteintech), Anti-Lipoic Acid (1:1000; ab58724, Abcam), FDX1 (1:1000; DF7950, Affinity), DLAT (1:1000; DF14046, Affinity), and GAPDH (1:3000; 10494-1-AP, Proteintech).

***Mice experiments***

We generated NCI-H23 cell-derived xenografts (CDXs) to explore the antitumor effects of CuNPs, AMG-510, and the combination of CuNPs with AMG-510 in BALB/c nude mice (Shanghai SLAC Laboratory Animal Co., Ltd., Shanghai, China). NCI-H23 cells (5×10^6^ cells/100 μL) were injected subcutaneously after one week of feeding. The mice were randomly divided into four groups (control, CuNPs, AMG-510, and combination of CuNPs and AMG-510) when the tumor volume was approximately 50-135 mm^3^. The mice in the control group did not receive any drug; the mice in the CuNPs group were administered 100 μg/100 μl CuNPs by intraperitoneal injection on alternate day; the mice in the AMG-510 group were administered 30 mg/kg AMG-510 by gavage (100 μl suspension) every day; and the mice in the combination group were administered 100 μg/100 μl CuNPs by intraperitoneal injection and 30 mg/kg AMG-510 by gavage (100 μl suspension). Sodium carboxymethylcellulose (0.5%, TargetMol Co. Ltd., USA) was used as the resuspension medium for AMG-510 to obtain a stable homogeneous mixture. The mice were weighed at regular intervals. Twenty-eight days after the first drug injection, differences in the tumor size were evident among the groups, and the animal experiment was ended. After the mice were anesthetized, they were killed via cervical dislocation. Then, the tumors were excised and weighed, and the tumor tissue was preserved.

***Hematoxylin-eosin (HE) and immunohistochemical staining***

CDX tumor tissues, heart, lung, spleen, kidney, and liver were fixed with 4% paraformaldehyde, embedded in paraffin, cut into sections (4 μm), and subjected to HE staining. The sections were observed under a microscope to assess histopathological changes in the cortex. Staining of paraffin-embedded CDX tumor tissues was performed with an EnVision system according to the manufacturer’s instructions^2^ (Glostrup, Dako, Denmark). A recombinant anti-Ki67 antibody (1:1000; ab279653; Abcam Co. Ltd., UK) was used as the primary antibody. Antibodies in the Dako REAL EnVision Detection System were used as secondary antibodies.

***TUNEL staining***

Cell passaging was performed on tumor tissue after dewaxing and hydration, followed by TUNEL staining (TUNEL BrightRed Apoptosis Detection Kit, Vazyme, A113-03), and sample washing after staining.

***mRNA sequencing***

Total RNA was extracted using TRIzol reagent (Invitrogen, CA, USA) according to the manufacturer’s protocol (https://www.thermofisher.cn/cn/zh/home/brands/product-brand/trizol.html). RNA purity and concentration were evaluated using a NanoDrop 2000 spectrophotometer (Thermo Scientific, USA). RNA integrity was assessed using an Agilent 2100 Bioanalyzer (Agilent Technologies, Santa Clara, CA, USA). Then, libraries were constructed using the VAHTS Universal V6 RNA-seq Library Prep Kit according to the manufacturer’s instructions^3^. The libraries were sequenced on the Illumina NovaSeq 6000 platform, and 150 bp paired-end reads were generated. Approximately 50 million raw reads were generated for each sample^3^. The raw reads in fastq format were first processed using fastp, and the low-quality reads were removed to obtain the clean reads. Then, approximately 40M clean reads per sample were retained for subsequent analyses. Finally, the clean reads were mapped to the reference genome using HISAT2^3^. The data of RNA sequencing was available at public repositories (https://ngdc.cncb.ac.cn/omix/view/OMIX 012657).

***RNA extraction and qRT-PCR***

RNA was extracted from NCI-H23 and SW-1573 cells using TRIzol reagent (Invitrogen, CA, USA), and 1 μg of mRNA was used to synthesize cDNA using HiScript III RT SuperMix for qPCR (Vazyme, R323-01). qRT-PCR was performed using ChamQ Universal SYBR qPCR Master Mix (Vazyme, Q711-02) according to a standard protocol (https://bio.vazyme.com/product/167.html). For each treatment group, three biological replicates were collected. The sequences of the primers used for amplification of targeted gene were detailed in **Table S1**. Gene expression fold changes were calculated using the comparative CT method (2 ^− ΔΔCT^), and the expression value in untreated human trabecular meshwork cells was set to 1 as the normalization control. The housekeeping gene GAPDH was used as the endogenous control gene in these experiments.

***Statistical analysis***

GraphPad Prism 9 software was used for statistical analysis. All experiments were repeated at least twice. Differential expression analysis was performed using DESeq2. A *p* value < 0.05 and a foldchange > 2 or < -2 were set as the threshold criteria for the identification of significantly DEGs^2, 4, 5^. The bioinformatic analyses were performed in a online tool (https://cloud.oebiotech.com/#/home). The student’ t test was used to determine the significance of differences in the tumor weight between the two groups. The standard deviation (SD) was calculated to evaluate the stability of the data. The Kaplan-Meier analysis and Log-rank test were used in this study. A two-sided *p* value < 0.05 was considered to indicate statistical significance. **p* < 0.05; ***p* < 0.01; ****p* < 0.001; *****p* < 0.0001; ns, not significant.

**Supplementary Figures**

**Figure. S1**. Hydrodynamic size of CuNPs at 1-, 7- and 14-day period in phosphate buffer solution (PBS) and fetal bovine serum (FBS).

**Figure. S2**. **The results of cell cycle experiments showed that in SW-1573 cell lines.** Flow cytometric analysis of SW-1573 cells treated with PBS, 5.8 ppm, and 58.3 ppm CuNPs (n = 3, mean ± SD). Student’s t-test was performed: ^*^*p* < 0.05, ^**^*p* < 0.01, ^***^*p* < 0.001.

**Figure. S3**. **The results of cell cycle experiments showed that in NCI-H23 cell lines.** Flow cytometric analysis of NCI-H23 cells treated with PBS, 5.8 ppm, and 58.3 ppm CuNPs (n = 3, mean ± SD). Student’s t-test was performed: ^*^*p* < 0.05, ^**^*p* < 0.01, ^***^*p* < 0.001.

**Figure. S4**. The significant differences in the expression of cuproptosis-related genes in SW-1573 cell line.

**Figure. S5**. The significant differences in the expression of cuproptosis-related genes in NCI-H23 cell line.

 **Figure. S6.** Immunofluorescence assay of the level of intracellular reactive oxygen species (ROS) after treatment of 24h CuNPs at different concentrations.

**Figure. S7.** **Ferrostatin-1 (2 μM) to determine whether CuNPs could induce ferroptosis in LUAD cells.** (A). treatment of RSL3; (B). treatment of CuNPs; (C). the comparison between CuNPs (5.8 ppm) and CuNPs (5.8 ppm) plus Ferrostatin-1 (2 μM). Student’s t-test was performed: ^*^*p* < 0.05, ^**^*p* < 0.01, ^***^*p* < 0.001.

**Figure. S8.** Necrosulfonamide (2 μM), Z-VAD-FMK (2 μM), and Necrostatin-2 racemate (2 μM) to determine whether CuNPs (5.8 ppm) could induce necrosis in SW-1573 (A) and NCI-H23 (B) cell lines. Cisplatin (1 μM) is a positive control. Student’s t-test was performed: ^*^*p* < 0.05, ^**^*p* < 0.01, ^***^*p* < 0.001.

**Figure. S9**. The effect of different does of AMG-510 in LUAD cells. Student’s t-test was performed: ^*^*p* < 0.05, ^**^*p* < 0.01, ^***^*p* < 0.001.

**Figure. S10.** The effect of different does of CuNPs in NCI-H358 cells.

**Figure. S11**. The effect of the combination of CuNPs and AMG-510 by orthogonal experiments in SW-1573 cell lines. For example, when AMG-510 is not added, the inhibition rate of 0.12 ppm CuNPs is 7%; in the absence of CuNPs, the inhibition rate of 2.5 μM AMG-510 was 17%. However, when 0.12 ppm CuNPs and 2.5 μM AMG-510 were added at the same time, the inhibition rate reached 29%, which was higher than 24% (sum of 7%+17%).

**Figure. S12.** The effect of the combination of CuNPs and AMG-510 by orthogonal experiments in NCI-H23 cell lines. For example, when AMG-510 is not added, the inhibition rate of 0.12 ppm CuNPs is 15%; in the absence of CuNPs, the inhibition rate of 2.5 μM AMG-510 was 6.6%. However, when 0.12 ppm CuNPs and 2.5 μM AMG-510 were added at the same time, the inhibition rate reached 35%, which was higher than 21.3% (sum of 15%+6.6%).

**Figure. S13.** The effect of the combination of AMG-510 and Cu-MOF in NCI-H23 (A) and SW-1573 cell lines.

**Figure. S14.** The possible resistance targets of ARS-853 using genome-wide CRISPR-Cas9.

**Figure. S15.** The expression level of ATP7B in the three cell lines by western blot.

**Figure. S16**. The expression level of ATP7B in wild-type NCI-H358 and ATP7B-overexpression cell lines by western blot.

**Figure. S17**. The effect of different does of AMG-510 in NCI-H358-OE and NCI-H358 cells. OE: *ATP7B*-over expression.

**Figure. S18**. *ATP7B* regulates *VEGFA*/*PDGFB* expression. KEGG analyses in the comparison between NCI-H23 and NCI-H358 (A); KEGG analyses in the comparison between SW-1573 and NCI-H358 (B); KEGG analyses in the comparison between *ATP7B* low-expression and high-expression patients (C); KEGG analyses in the comparison between *ATP7B-*knockout and wild type cell lines (D). Cluster analysis of calcium signaling pathway in NCI-H358 *vs.* SW-1573 (E) and NCI-H358 *vs.* NCI-H23 (F) comparisons.

 **Figure. S19.** Substantial view of each organ (A); change in body weight of mice (B); HE staining of each organ (C). One-way analysis of variance (ANOVA) was performed: ^***^*p* < 0.001, ^**^*p* < 0.01, ^*^*p* < 0.05.

**Supplementary Tables**

**Table S1. The primer sequence of targeted genes.**

| **Gene** | **Primer sequence (5' -> 3')** | |
| --- | --- | --- |
|  | Forward Primer | Reverse Primer |
| human  *ATP7B* | GCCAGCATTGCAGAAGGAAAG | TGATAAGTGATGACGGCCTCT |
| human  *LIPT1* | CCTCTGTTGTAATTGGTAGGCAT | CTGGGGTTGGACAGCATTCAG |
| human  *LIPT2* | CTGACGCCCGAGGAAACTG | GTGGGATGTGATGTGCCTTC |
| human  *LIAS* | CAGCCCAGTCAGACCGTTAAG | TTTCTGGCGTTTTAGGTTTCCT |
| human  *DLD* | CTCATGGCCTACAGGGACTTT | GCATGTTCCACCAAGTGTTTCAT |
| human  *ACSM1* | GGGGCATCCACAAATCCTTC | TCTTGGGGCTCCAAATTCTGA |
| human  *GAPDH* | GGAGCGAGATCCCTCCAAAAT | GGCTGTTGTCATACTTCTCATGG |
| human  *ATP7B* | Sense | Antisense |
| si-1 | GGAAUUGGGUGCAAAGUCA | UGACUUUGCACCCAAUUCC |
| si-2 | GUGCAUCCUGUGUGUCUAA | UUAGACACACAGGAUGCAC |
| si-3 | CCAAUUGAUAUUGAGCGGUUA | UAACCGCUCAAUAUCAAUUGG |

**Table S2. The calculation of the combination index (CI) of combining AMG-510 with CuNPs in NCI-H23 cell line.**

| **BlockID** | **Drug1** | **Drug2** | **Conc1 (ppm)** | **Conc2 (μM)** | **CI** | **Response (%)** |
| --- | --- | --- | --- | --- | --- | --- |
| 1 | CuNPs | AMG-510 | 0 | 0 | - | 0 |
| 1 | CuNPs | AMG-510 | 0.12 | 0 | - | 0 |
| 1 | CuNPs | AMG-510 | 0 | 0.001 | - | 1.24 |
| 1 | CuNPs | AMG-510 | 0.12 | 0.001 | 0 | 1.18 |
| 1 | CuNPs | AMG-510 | 0 | 0.003 | - | -0.82 |
| 1 | CuNPs | AMG-510 | 0.12 | 0.003 | 0 | 4.98 |
| 1 | CuNPs | AMG-510 | 0 | 0.01 | - | 3.98 |
| 1 | CuNPs | AMG-510 | 0.12 | 0.01 | 0 | 9.93 |
| 1 | CuNPs | AMG-510 | 0 | 0.1 | - | 0.53 |
| 1 | CuNPs | AMG-510 | 0.12 | 0.1 | 0.01 | 32.35 |
| 1 | CuNPs | AMG-510 | 0 | 0.3 | - | 1.33 |
| 1 | CuNPs | AMG-510 | 0.12 | 0.3 | 0.02 | 37.49 |
| 1 | CuNPs | AMG-510 | 0 | 1 | - | 3.15 |
| 1 | CuNPs | AMG-510 | 0.12 | 1 | 0.06 | 44.08 |
| 1 | CuNPs | AMG-510 | 0 | 3 | - | 9.12 |
| 1 | CuNPs | AMG-510 | 0.12 | 3 | 0.27 | 51.1 |
| 1 | CuNPs | AMG-510 | 0 | 5 | - | 27.13 |
| 1 | CuNPs | AMG-510 | 0.12 | 5 | 0.36 | 68.47 |
| 1 | CuNPs | AMG-510 | 0 | 10 | - | 54.23 |
| 1 | CuNPs | AMG-510 | 0.12 | 10 | 0.44 | 83.61 |
| 1 | CuNPs | AMG-510 | 0 | 30 | - | 71.01 |
| 1 | CuNPs | AMG-510 | 0.12 | 30 | 0.59 | 86.23 |
| 1 | CuNPs | AMG-510 | 0 | 50 | - | 86.3 |
| 1 | CuNPs | AMG-510 | 0.12 | 50 | 0.91 | 87.65 |

CI = 1: additive effect, CI < 1: synergy effect, CI > 1: antagonistic effect

**Table S3. The calculation of the combination index (CI) of combining AMG-510 with CuNPs in SW-1573 cell line.**

| **BlockID** | **Drug1** | **Drug2** | **Conc1 (ppm)** | **Conc2 (μM)** | **CI** | **Response (%)** |
| --- | --- | --- | --- | --- | --- | --- |
| 1 | CuNPs | AMG-510 | 0 | 0 | - | 0 |
| 1 | CuNPs | AMG-510 | 0.12 | 0 | - | 0 |
| 1 | CuNPs | AMG-510 | 0 | 0.001 | - | -1.62 |
| 1 | CuNPs | AMG-510 | 0.12 | 0.001 | 0 | 0.14 |
| 1 | CuNPs | AMG-510 | 0 | 0.003 | - | 0.21 |
| 1 | CuNPs | AMG-510 | 0.12 | 0.003 | 0 | 1.76 |
| 1 | CuNPs | AMG-510 | 0 | 0.01 | - | -1.4 |
| 1 | CuNPs | AMG-510 | 0.12 | 0.01 | 0 | 16.24 |
| 1 | CuNPs | AMG-510 | 0 | 0.1 | - | -1.42 |
| 1 | CuNPs | AMG-510 | 0.12 | 0.1 | 0.01 | 28.42 |
| 1 | CuNPs | AMG-510 | 0 | 0.3 | - | -0.8 |
| 1 | CuNPs | AMG-510 | 0.12 | 0.3 | 0.02 | 28.21 |
| 1 | CuNPs | AMG-510 | 0 | 1 | - | -0.94 |
| 1 | CuNPs | AMG-510 | 0.12 | 1 | 0.04 | 33.93 |
| 1 | CuNPs | AMG-510 | 0 | 3 | - | 0.3 |
| 1 | CuNPs | AMG-510 | 0.12 | 3 | 0.19 | 41.9 |
| 1 | CuNPs | AMG-510 | 0 | 5 | - | 9.74 |
| 1 | CuNPs | AMG-510 | 0.12 | 5 | 0.27 | 61.71 |
| 1 | CuNPs | AMG-510 | 0 | 10 | - | 26.68 |
| 1 | CuNPs | AMG-510 | 0.12 | 10 | 0.35 | 81.85 |
| 1 | CuNPs | AMG-510 | 0 | 30 | - | 51.52 |
| 1 | CuNPs | AMG-510 | 0.12 | 30 | 0.46 | 86.11 |
| 1 | CuNPs | AMG-510 | 0 | 50 | - | 76.46 |
| 1 | CuNPs | AMG-510 | 0.12 | 50 | 0.69 | 88.59 |

CI = 1: additive effect, CI < 1: synergy effect, CI > 1: antagonistic effect

**Reference**

1.	Wei, C. et al. Nuclear mitochondrial acetyl-CoA acetyltransferase 1 orchestrates natural killer cell-dependent antitumor immunity in colorectal cancer. *Signal Transduct Target Ther* **10**, 138 (2025).

2.	Li, K. et al. Apoptosis and cuproptosis Co-activated Copper-based metal-organic frameworks for cancer therapy. *Journal of nanobiotechnology* **22**, 546 (2024).

3.	Chen, S., Zhou, Y., Chen, Y. & Gu, J. fastp: an ultra-fast all-in-one FASTQ preprocessor. *Bioinformatics (Oxford, England)* **34**, i884-i890 (2018).

4.	Wu, L.-L. et al. AMG-510 and cisplatin combination increases antitumor effect in lung adenocarcinoma with mutation of KRAS G12C: a preclinical and translational research. *Discover Oncology* **14** (2023).

5.	Li, K. et al. The characterization of tumor microenvironment infiltration and the construction of predictive index based on cuproptosis-related gene in primary lung adenocarcinoma. *Front Oncol* **12**, 1011568 (2022).
